# Estimating the future clinical and economic benefits of improving osteoporosis diagnosis and treatment among postmenopausal women across eight European countries

**DOI:** 10.1007/s11657-023-01230-0

**Published:** 2023-05-16

**Authors:** Eric J. Yeh, Matthew Gitlin, Francesc Sorio, Eugene McCloskey

**Affiliations:** 1 Amgen Inc., Thousand Oaks, CA USA; 2 BluePath Solutions, Inc., Los Angeles, CA USA; 3 Amgen (Europe) GmbH, Risch-Rotkreuz, Switzerland; 4 Centre for Metabolic Bone Diseases, University of Sheffield, Beech Hill Road, Sheffield, UK; 5 Centre for Integrated Research in Musculoskeletal Ageing, University of Sheffield, Sheffield, UK; 6 Mellanby Centre for Musculoskeletal Research, Healthy Lifespan Institute (HELSI), University of Sheffield, Sheffield, UK

**Keywords:** Adherence, Assessment, Europe, Fracture, Osteoporosis, Treatment

## Abstract

**Summary:**

A population-level, cross-sectional model was developed to estimate the clinical and economic burden of osteoporosis among women (≥ 70 years) across eight European countries. Results demonstrated that interventions aimed at improving fracture risk assessment and adherence would save 15.2% of annual costs in 2040.

**Purpose:**

Osteoporosis is associated with significant clinical and economic burden, expected to further increase with an ageing population. This modelling analysis assessed clinical and economic outcomes under different hypothetical disease management interventions to reduce this burden.

**Methods:**

A population-level, cross-sectional cohort model was developed to estimate numbers of incident fractures and direct costs of care among women (≥ 70 years) in eight European countries under different hypothetical interventions: (1) an improvement in the risk assessment rate, (2) an improvement in the treatment adherence rate and (3) a combination of interventions 1 and 2. A 50% improvement from the status quo, based on existing disease management patterns, was evaluated in the main analysis; scenario analyses evaluated improvement of either 10 or 100%.

**Results:**

Based on existing disease management patterns, a 44% increase in the annual number of fractures and costs was predicted from 2020 to 2040: from 1.2 million fractures and €12.8 billion in 2020 to 1.8 million fractures and €18.4 billion in 2040. Intervention 3 provided the greatest fracture reduction and cost savings (a decrease of 17.9% and 15.2% in fractures and cost, respectively) in 2040 compared with intervention 1 (decreases of 8.7% and 7.0% in fractures and cost, respectively) and intervention 2 (10.0% and 8.8% reductions in fracture and cost, respectively). Scenario analyses showed similar patterns.

**Conclusion:**

These analyses suggest that interventions which improve fracture risk assessment and adherence to treatments would relieve the burden of osteoporosis, and that a combination strategy would achieve greatest benefits.

**Supplementary Information:**

The online version contains supplementary material available at 10.1007/s11657-023-01230-0.

## Introduction

Osteoporosis is a chronic condition characterised by low bone mineral density (BMD) and deterioration of bone tissue, leading to increased susceptibility to fracture [1]. It primarily occurs in postmenopausal women [2]. It was estimated that 22% of women aged 50 years or older in 27 countries of the European Union (EU) plus the UK and Switzerland had osteoporosis in 2019 [3]. Furthermore, given that the prevalence of osteoporosis increases with age, the clinical and economic burden of osteoporosis in countries with ageing populations is expected to rise considerably over the coming decades [2, 4]. A recent study analysing the burden and management of fragility fractures in France, Germany, Italy, Spain, Sweden and the UK estimated that the total numbers of fractures for both men and women will increase by 23% in the next decade, from 2.7 million in 2017 to 3.3 million in 2030 [4]. Over the same period, annual fracture costs are expected to increase by 27%, from €37.5 billion to €47.4 billion [4].

Optimal diagnosis and treatment of osteoporosis, in line with clinical practice guidelines [5, 6], may help reduce this significant burden on healthcare systems. However, real-world data highlight that osteoporosis remains under-diagnosed and untreated [4, 7]. Results from a large, cross-sectional, observational European study showed that over two-thirds (68%) of women aged 70 years or older who had an increased risk of fracture had not been diagnosed with osteoporosis by their primary care physician, and three-quarters (75%) were not receiving any anti-osteoporosis medication [7]. These findings are supported by the results of a separate study conducted in France, Germany, Italy, Spain, Sweden and the UK, which estimated the osteoporosis treatment gap (i.e. the proportion of eligible women not receiving treatment with osteoporosis drugs) to be 73% in 2017 [4].

In addition to addressing diagnosis and treatment gaps, improving patient adherence to anti-osteoporosis medications may also help reduce the high burden of osteoporosis and fractures. An expert group of the European Society for Clinical and Economic Aspects of Osteoporosis, Osteoarthritis and Musculoskeletal Diseases and the International Osteoporosis Foundation determined that medication non-adherence is associated with an increased risk of fracture (for example, relative risk of 1.28 for hip fractures and 1.43 for vertebral fractures) [8], leading to a substantial decrease in the clinical and economic benefits of drug therapy [9]. This is an important consideration, as a systematic review of factors affecting medication adherence among patients with osteoporosis reported a wide variation in medication adherence rates, with some as low as 13% or as high as 95% [10]. Overall, the increasing clinical and economic burden of osteoporosis, combined with suboptimal diagnosis and under-utilised anti-osteoporosis medications, represents an urgent unmet need, and improvements in the management of this chronic condition are warranted.

The primary aim of this modelling analysis was to compare projected clinical and economic outcomes for osteoporosis under different hypothetical interventions. Various rates of risk assessment and adherence to anti-osteoporosis medications were used to assess whether these interventions would lead to net benefits on numbers of incident fractures and total costs of care. These interventions could then be used to recommend optimal management approaches. Eight European countries (Belgium, France, Germany, Ireland, Poland, Slovakia, Switzerland and the UK) were included and modelled in these analyses.

## Methods

Using previously published data and modelling approaches [7, 11], a population-level, cross-sectional cohort model was developed to estimate the clinical and economic burden of osteoporosis (specifically, the annual incidence of osteoporotic fractures and related costs of care). The study populations were women aged 70 years or older across eight European countries. The model predicted outcomes over a 20-year period (from 2020 to 2040) under different hypothetical interventions with various sets of improvement from the status quo, which was based on existing disease management patterns reported by McCloskey et al. [7]. The three hypothetical interventions were assessed: intervention 1, an improvement in risk assessment rate (which would have a subsequent impact on the number of patients receiving anti-osteoporosis medications); intervention 2, a reduction in rates of non-adherence to anti-osteoporosis medications and intervention 3, a combination of interventions 1 and 2.

### Model overview

The cohort model was based on a previously published microsimulation approach [11]. Details regarding this model, its inputs, validation and additional data sources can be found in Table 1 and in the Supplementary Material (Supplementary Methods; Supplementary Tables S1–S2). Overall, the model estimation of the clinical and economic burden associated with osteoporosis (i.e., annual numbers of fractures and costs of care as key model outcomes) involved four distinct steps.

**Table 1:** Key fracture and treatment model inputs

	Distribution of fractures, %			Efficacy versus placebo (RR) weighted based on treatment mix			Fracture cost, €			Anti-osteoporosis medication cost^a^, €	BMD measurement, €	Status quo treatment rate among women ≥ 70^a^, %	Identification rate of those at risk among those assessed, %^a^	Treatment rate among those at risk, %^b^
Country	H	V	NHNV	H	V	NHNV	H	V	NHNV					
Belgium	21	14	65	0.62	0.46	0.83	13,381	2959	4932	216	34	22	57	33
France	21	14	64	0.55	0.43	0.71	13,286	3312	7398	151	41	14	69	18
Germany	20	15	65	0.60	0.45	0.82	19,218	5585	9063	305	36	6	58	9
Ireland	19	14	67	0.60	0.48	0.82	16,247	3594	5996	252	99	29	48	47
Poland	19	15	65	0.60	0.52	0.83	5606	1241	1950	85	10	8	51	12
Slovakia	18	15	67	0.62	0.47	0.83	4690	1037	2047	173	32	13	41	25
Switzerland	21	15	65	0.59	0.40	0.80	17,954	11,484	12,063	468	98	27	76	36
UK	16	12	72	0.61	0.56	0.84	11,055	2756	6334	36	51	20	49	35

*BMD* bone mineral density, *H* hip, *NHNV* non-hip, non-vertebral, *RR* relative risk, *V* vertebral

^a^The anti-osteoporosis treatments included were based on the treatments utilised within the study conducted by McCloskey et al*.* The therapies included oral bisphosphonate (which was assumed to be alendronate), denosumab, zoledronate, teriparatide and raloxifene

^b^Using these data, new treatment rates were calculated as: new treatment rate = treatment_Existing_ + (assessment_new_ − assessment_Existing_) × % of assessed identified as at risk × % at risk that are treated

#### 1. Estimation of fracture risk under the status quo

The first step estimated the risk of fractures (hip, vertebral or non-hip, non-vertebral) under the status quo. The estimation used a simplified version of the Fracture Risk Assessment Tool (FRAX) [12], which was populated with country-level demographic and risk factor data from the literature, publicly available databases and online sources (Table 1 and Supplementary Tables S1–S2). The model only permitted an individual to record up to one fracture per year for each fracture type.

#### 2. Estimation of fracture risk under different hypothetical interventions

The second step estimated the reduction in fracture risk that would occur with use of anti-osteoporosis medications in each of the three hypothetical interventions. The following treatments were included: oral bisphosphonate (which was assumed to be alendronate), denosumab, zoledronate, teriparatide and raloxifene. The distribution of anti-osteoporosis medications at the country level and corresponding treatment efficacy data, obtained from the literature (Table 1 and Supplementary Table S1 and Table S3), was used to generate a country-specific population fracture risk. Treatment efficacy for the included osteoporosis therapies was extracted from a network meta‐analysis of clinical trials that examined the comparative effectiveness of various available pharmacological osteoporosis therapies [13] (Supplementary Table S3). New treatment rates were computed based on the formula included in the footnote of Table 1. The fracture risk was estimated for each intervention by country and year.

#### 3. Estimation of annual number of fractures

The third step estimated the number of fractures based on the distributions of fracture types, estimated risk of each fracture type and the size of the population at risk. The total number of fractures was estimated for each intervention by country and year.

#### 4. Estimation of annual costs of care

The last step estimated associated costs of care. Costs of osteoporotic fractures, treatments and risk assessment are presented in Supplementary Table S1. Fracture costs were estimated using first year direct medical costs for each fracture type multiplied by the estimated number of fractures from step 3. All costs were converted to 2020 euros, adjusted for inflation using the appropriate consumer price index in each respective country. A weighted average of treatment costs was estimated based on treatment rates, treatment costs, treatment types and market share data. Risk assessment was based on BMD-associated FRAX charts which include a combination of BMD and the prevalence of specific clinical risk factors. Risk assessment costs were estimated by multiplying the unit cost of a BMD measurement in each country by the rate of risk assessment specified in each intervention.

### Analysis

Outcomes analyses were conducted by country and by year (2020, 2025, 2030, 2035 and 2040) and the results presented overall and by individual country. To facilitate comparisons and identify trends across countries (where different health systems would lead to varying hypothetical interventions, costs, treatment availabilities, etc.), numbers of fractures and cost data are presented per 100,000 population and percent change in addition to absolute values.

The main analysis was performed to examine a 50% improvement in risk assessment rates, and/or an improvement in adherence rate by reducing 50% of the non-adherence part from the status quo (termed intervention 1_50_, 2_50_ and 3_50_, respectively, Supplemental Table S4). For example, if the original adherence rate was 40% in the status quo, non-adherence was 60%. Therefore, a 50% reduction would yield a 30% increase in the new adherence rate from 40 to 70% in the hypothetical intervention 2_50_. These rates used in the main analysis are summarised in Supplementary Table S5. The new treatment rates were calculated based on the information in Table 1. It was assumed that in the status quo, the risk assessment rate was 25%, and the adherence rate to all anti-osteoporosis medications was 40% across all countries (derived from references in Supplementary Table S1). When intervention 3 was assessed, a hierarchical approach was applied: the estimated population treated was first assessed based on an improvement in risk assessment rates, and then the reduction in non-adherence rates was considered. The impact of each hypothetical intervention was calculated as differences in outcomes (for example, numbers of fractures or total costs of care) from the status quo.

Scenario analyses were also performed to examine either 10 or 100% improvements in risk assessment and 10 or 100% reductions in non-adherence rates for each intervention (termed as Scenario 1_10_, 2_10_, 3_10_, 1_100_, 2_100_ or 3_100_, respectively, Supplemental Table S7).

## Results

### Burden of disease under the status quo

The annual projected population sizes for the eight European countries, the projected number of osteoporotic fractures and total costs of care under the status quo are presented in Table 2. Overall, the number of women 70 years of age or older in eight European countries was projected to increase by 40% between 2020 and 2040 (to 33 million), the annual number of fractures was projected to increase by 44% (to 1.8 million), and the associated annual medical costs were projected to increase by 44% (to €18.4 billion).

**Table 2:** Projected population size, annual number of fractures and total costs of care under the status quo

Country	2020	2025	2030	2035	2040	Ratio (2040 to 2020)
Annual population sizes in women aged ≥ 70 years, *n*						
Belgium	909,965	981,569	1,079,222	1,189,198	1,272,290	1.40
France	5,755,944	6,417,296	7,071,131	7,721,280	8,246,896	1.43
Germany	7,617,401	8,004,192	8,540,931	9,441,730	10,266,513	1.35
Ireland	266,580	316,146	370,709	430,199	487,401	1.83
Poland	2,808,079	3,313,821	3,812,156	4,025,639	4,076,774	1.45
Slovakia	362,914	432,737	495,271	537,088	558,324	1.54
Switzerland	665,515	724,290	798,327	899,196	996,235	1.50
UK	5,082,343	5,475,642	5,914,422	6,475,365	6,911,825	1.36
Total	23,468,741	25,665,693	28,082,169	30,719,695	32,816,258	1.40
Annual numbers of osteoporotic fractures, *n*						
Belgium	48,700	51,500	56,597	63,674	70,190	1.44
*n*/100,000 population	5352	5247	5244	5354	5517	
France	303,133	328,918	370,321	420,066	462,294	1.53
*n*/100,000 population	5266	5125	5237	5440	5606	
Germany	409,939	429,444	447,562	484,442	541,024	1.32
*n*/100,000 population	5382	5365	5240	5131	5270	
Ireland	14,768	17,469	20,952	24,642	28,494	1.93
*n*/100,000 population	5540	5526	5652	5728	5846	
Poland	85,578	95,823	111,468	128,478	140,632	1.64
*n*/100,000 population	3048	2892	2924	3192	3450	
Slovakia	16,868	19,586	23,181	26,279	28,028	1.66
*n*/100,000 population	4648	4526	4681	4893	5020	
Switzerland	50,952	56,590	63,088	70,596	78,878	1.55
*n*/100,000 population	7656	7813	7903	7851	7918	
UK	308,381	339,412	372,077	406,832	434,760	1.41
*n*/100,000 population	6068	6199	6291	6283	6290	
Total	1,238,320	1,338,742	1,465,247	1,625,008	1,784,301	1.44
*n*/100,000 population	5276	5216	5218	5290	5437	
Annual costs of care, in millions						
Belgium	€343	€365	€401	€448	€494	1.44
*n*/100,000 population	€37.7	€37.2	€37.2	€37.7	€38.8	
France	€4628	€5026	€5654	€6399	€7042	1.52
*n*/100,000 population	€80.4	€78.3	€80.0	€82.9	€85.4	
Germany	€4473	€4686	€4883	€5304	€5903	1.32
*n*/100,000 population	€58.7	€58.6	€57.2	€56.2	€57.5	
Ireland	€132	€155	€185	€218	€250	1.89
*n*/100,000 population	€49.7	€49.1	€50.0	€50.6	€51.3	
Poland	€235	€264	€307	€352	€383	1.63
*n*/100,000 population	€8.4	€8.0	€8.0	€8.7	€9.4	
Slovakia	€48	€56	€65	€74	€78	1.64
*n*/100,000 population	€13.2	€13.0	€13.2	€13.7	€14.0	
Switzerland	€744	€824	€918	€1028	€1148	1.54
*n*/100,000 population	€111.9	€113.8	€115.0	€114.3	€115.3	
UK	€2187	€2405	€2634	€2880	€3078	1.41
*n*/100,000 population	€43.0	€43.9	€44.5	€44.5	€44.5	
Total	€12,790	€13,782	€15,047	€16,701	€18,376	1.44
*n*/100,000 population	€54.5	€53.7	€53.6	€54.4	€56.0	

*n*/100,000 population is based on women aged ≥ 70 years

### Impact of three interventions on numbers of fractures and costs of care in the main analysis

**Fig. 1:**
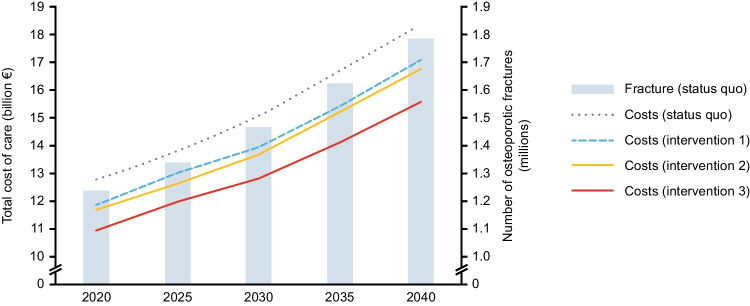
Projected annual burden of osteoporotic fractures in the main analysis for eight European countries

Figure 1 shows predicted annual costs of care and annual number of fractures from 2020 to 2040 under the status quo and three hypothetical interventions. Table 3 shows differences in projected annual numbers of fractures, annual costs of care and the relative percentage reduction from the status quo under each hypothetical intervention. While projected results from all three hypothetical interventions show potential net benefits over the status quo in the main analysis, intervention 3_50_ resulted in greater net benefits (319,191 fractures prevented and €2.8 billion savings) in 2040 than intervention 1_50_ (154,548 fractures prevented; €1.3 billion savings) or intervention 2_50_ (179,265 fractures prevented; €1.6 billion savings). The patterns are similar in 2025, 2030 and 2035.

**Table 3:** Potential net benefit by type of hypothetical intervention for eight European countries

Net benefits from status quo (relative percentage reduction)		2020	2025	2030	2035	2040
Main analysis						
Intervention 1_50_ (50% improvement in risk assessment rates)	Fractures	− 114,688 (− 9.3%)	− 98,933 (− 7.4%)	− 135,125 (− 9.2%)	− 147,302 (− 9.1%)	− 154,548 (− 8.7%)
	Costs, in millions	− €938 (− 7.3%)	− €772 (− 5.6%)	− €1110 (− 7.4%)	− €1263 (− 7.6%)	− €1284 (− 7.0%)
Intervention 2_50_ (50% reduction in non-adherence)	Fractures	− 126,909 (− 10.2%)	− 133,958 (− 10.0%)	− 151,884 (− 10.4%)	− 167,808 (− 10.3%)	− 179,265 (− 10.0%)
	Costs, in millions	− €1103 (− 8.6%)	− €1138 (− 8.3%)	− €1362 (− 9.1%)	− €1490 (− 8.9%)	− €1609 (− 8.8%)
Intervention 3_50_ (50% improvement in both risk assessment and non-adherence)	Fractures	− 217,843 (− 17.6%)	− 218,447 (− 16.3%)	− 264,672 (− 18.1%)	− 300,179 (− 18.5%)	− 319,191 (− 17.9%)
	Costs, in millions	− €1850 (− 14.5%)	− €1815 (− 13.2%)	− €2235 (− 14.9%)	− €2579 (− 15.4%)	− €2797 (− 15.2%)
Scenario analysis						
Intervention 1_10_ (10% improvement in risk assessment rates)	Fractures	− 64,918 (− 5.2%)	− 69,906 (− 5.2%)	− 88,980 (− 6.1%)	− 101,355 (− 6.2%)	− 105,660 (− 5.9%)
	Costs, in millions	− €560 (− 4.4%)	− €640 (− 4.6%)	− €825 (− 5.5%)	− €921 (− 5.5%)	− €940 (− 5.1%)
Intervention 2_10_ (10% reduction in non-adherence)	Fractures	− 51,321 (− 4.1%)	− 42,547 (− 3.2%)	− 53,094 (− 3.6%)	− 68,269 (− 4.2%)	− 53,310 (− 3.0%)
	Costs, in millions	− €463 (− 3.6%)	− €338 (− 2.5%)	− €495 (− 3.3%)	− €631 (− 3.8%)	− €425 (− 2.3%)
Intervention 3_10_ (10% improvement in both risk assessment and non-adherence)	Fractures	− 96,213 (− 7.8%)	− 86,502 (− 6.5%)	− 113,394 (− 7.7%)	− 142,633 (− 8.8%)	− 150,239 (− 8.4%)
	Costs, in millions	− €862 (− 6.7%)	− €753 (− 5.5%)	− €998 (− 6.6%)	− €1323 (− 7.9%)	− €1388 (− 7.6%)
Intervention 1_100_ (100% improvement in risk assessment rates)	Fractures	− 160,618 (− 13.0%)	− 159,925 (− 11.9%)	− 187,280 (− 12.8%)	− 219,059 (− 13.5%)	− 262,216 (− 14.7%)
	Costs, in millions	− €1216 (− 9.5%)	− €1152 (− 8.4%)	− €1387 (− 9.2%)	− €1718 (− 10.3%)	− €2067 (− 11.2%)
Intervention 2_100_ (100% reduction in non-adherence)	Fractures	− 222,970 (− 18.0%)	− 232,778 (− 17.4%)	− 259,195 (− 17.7%)	− 285,643 (− 17.6%)	− 307,502 (− 17.2%)
	Costs, in millions	− €1994 (− 15.6%)	− €2035 (− 14.8%)	− €2333 (− 15.5%)	− €2505 (− 15.0%)	− €2737 (− 14.9%)
Intervention 3_100_ (100% improvement in both risk assessment and non-adherence)	Fractures	− 296,063 (− 23.9%)	− 307,237 (− 22.9%)	− 349,344 (− 23.8%)	− 389,970 (− 24.0%)	− 442,262 (− 24.8%)
	Costs, in millions	− €2330 (− 18.2%)	− €2356 (− 17.1%)	− €2751 (− 18.3%)	− €3050 (− 18.3%)	− €3447 (− 18.8%)

Projected reductions in annual costs of care in the main analysis for each individual country are presented in Supplementary Table S6. In the main analysis, projected cost reductions ranged from 19.2% (€221 million) in Switzerland to 5.4% (€4.2 million) in Slovakia for intervention 3_50_. Intervention 3_50_ would consistently provide greater cost savings than either intervention 1_50_ or intervention 2_50_ for individual countries.

### Impact of three interventions on numbers of fractures and costs of care in the scenario analyses

Scenario analyses showing the differences from the status quo in projected annual numbers of fractures and annual costs of care for each intervention scenario (i.e., intervention 1_10_, 2_10_, 3_10_, 1_100_, 2_100_ or 3_100_) for eight countries are also presented in Table 3. Data for individual countries are presented in Supplementary Table S6. Projected results were mostly consistent with those for the main analysis, with all interventions providing potential fracture reduction and cost savings over the status quo. With only 10% improvements in risk assessment and 10% reductions in non-adherence rates, intervention 3_10_ would lead to a greater cost saving (7.6% cost reduction of €1.4 billion) in 2040 than either intervention 1_10_ (5.1% cost reduction of €940 million) or intervention 2_10_ (2.3% cost reduction of €425 million). Similarly, intervention 3_100_ would provide a greater cost saving (18.8% cost reduction of €3.4 billion) than intervention 1_100_ (11.2% cost reduction of €2.1 billion) or intervention 2_100_ (14.9% cost reduction of €2.7 billion).

## Discussion

In this study, we developed a cross-sectional cohort model to project the annual clinical and economic burdens of osteoporotic fractures in women older than 70 years old across eight European countries over a 20-year period (2020–2040). This model was then used to assess the potential impact of various improved rates of risk assessment and/or adherence to anti-osteoporosis medications on these outcomes. Both the annual number of fractures and annual cost of care were predicted to increase by over 40% from 1.2 million fractures (€12.8 billion) in 2020 to 1.8 million fractures (€18.4 billion) in 2040 should the status quo remain. This is primarily due to a projected 40% increase in the number of women aged 70 years or older during this time frame, because it is reasonable to assume increased ageing populations in Europe. Results from both the main analysis and scenario analysis suggest interventions aimed at improving risk assessment and adherence to anti-osteoporosis medications would have a considerable impact in reducing the burden of osteoporotic fracture. In particular, interventions with 50% improved rates in risk assessment and adherence to anti-osteoporosis medications would lead to a 15.2% cost reduction, totalling nearly €2.8 billion for these eight European countries in 2040.

The substantial net benefits (i.e., reduction in numbers of fractures and cost savings) estimated by our model support the need for new policy measures and/or increased efforts to improve the assessment of osteoporotic fracture risk and adherence to anti-osteoporosis medications. The introduction of such interventions should ensure that more patients at high risk of osteoporosis are screened in a timely manner to diagnose osteoporosis. This would lead to initiation of appropriate anti-osteoporosis medications at the time when it is needed by the patient and advocate that patients remain adherent to treatment. In addition, high-quality data from registries of patients with osteoporosis and fragility fractures are needed to allow for vital national and local feedback. Importantly, considerations on how these data can be used to improve quality-of-care should be contemplated. Health education programmes for patients at risk of, or with, osteoporosis should also be considered to improve disease awareness, patient confidence and treatment adherence. Policy makers, payers and healthcare providers (e.g. primary care physicians) will be required to play key roles in the design and implementation of health education programmes for patients at risk of osteoporosis.

The results from our model are consistent with those of other similar analyses. However, unlike our model, the majority of previous studies only use data from individual countries. Previous studies also tend to present absolute values rather than estimate percentage changes in the number of fractures or total costs of care over time. Furthermore, these studies tend to focus on the impact of risk assessment without accounting for treatment adherence [4, 11, 14–16]. In our model, we have estimated fracture risk and the clinical and economic burden individually for each country using country-specific data, then aggregated to produce overall results. Thus, the results from this study could be considered more generalisable to the wider real-world population of patients than previous studies which only use data from individual countries. For the status quo, our model estimates are supported by a recent analysis conducted by Borgstrom et al., which estimated a 23% increase in fractures and a 27% increase in fracture-related costs in France, Germany, Italy, Spain, Sweden and the UK over a 13-year period [4]; our model estimates an approximate 40% increase over a 20-year period. With respect to the positive impact of improvements in risk assessment, studies in the USA, South Korea, Japan and China have all reported substantial reductions in clinical and economic burden, with estimated cost savings ranging from US$3.1 billion (Japan) to US$61.7 billion (China) over approximately 20-year periods [4, 11, 14–16]. The consistency of these findings across studies in different regions reflects the clear clinical and economic benefits associated with improvements in the management of osteoporosis.

As with any study, there are limitations that should be considered when interpreting the results. For example, while our population size projections considered dynamic changes in demographics, including age, over the model time horizon when estimating fracture risks using the FRAX tables, other risk factors such as smoking and prior fracture (which may also change over time) were not accounted for as separated model inputs in this analysis. Nevertheless, as the FRAX approach is still considered a reasonable and reliable estimation of fracture risk and is included in current osteoporosis diagnosis and management guidelines [5], the omission of some risk factor information would not be expected to have a significant impact on the final estimates. Furthermore, our model did not allow for individuals to incur multiple fractures of the same type within a single year, though it did allow an individual to experience up to three fractures in different locations (one each of hip, vertebral and non-hip, non-vertebral). This may lead to an underestimation of the potential burden of osteoporosis. Similarly, it was assumed that healthcare costs would remain constant, potentially underestimating projected spending growth. In addition, first year direct medical costs were used to estimate the annual costs of care. Substantial costs may occur after the first year of fracture with an increased probability of patients requiring nursing home care [17]. The model did not include costs related to the broader indirect economic and social burden of osteoporosis, such as lost productivity or reduced tax revenue as a result of productivity loss, paid and unpaid caregiver burden and home/environment adaptations. All these factors mean the model estimates are likely conservative. Indeed, in Europe, it has been shown that approximately one-third of costs related to osteoporosis are indirect, and considering the broader economic and social burdens of osteoporosis, this estimate of indirect costs is likely to be conservative [3]. Thus, the potential clinical and economic value of efforts to improve outcomes is likely to be greater than presented here. Another potential limitation of our analysis is the lack of consideration of adverse events associated with treatment. However, compared with the substantial burden associated with fractures, this would likely have minimal impact on the model results. An additional limitation was that vertebral fracture assessment was not included in our cost estimation. Studies have shown that vertebral fracture assessment may be cost effective when it is incorporated in routine screening for osteoporosis [18, 19]. Finally, the impact of increased treatment in men was not considered in our model. Osteoporosis also occurs in men aged 70 years or older [2]. It was estimated that 6.6% of men aged 50 years or older in 27 EU countries plus the UK and Switzerland had osteoporosis in 2019 [3]. Inclusion of men in this analysis would likely have increased the number of potential fractures prevented, and this is an area that should be explored in future research.

Regarding assumptions inherent in the model, assuming that all women aged 70 years or older had a BMD measurement when they underwent fracture risk assessment is likely a conservative approach, since less costly risk assessment methods, such as using clinical judgement or algorithms based on risks produced by FRAX, are also used in clinical practice. BMD measurement is often reserved for cases in which a treatment decision is uncertain. As such, potential cost savings based on improving risk assessment rates would likely be greater than those presented here. The model also assumed that the fracture reduction benefits of treatment in clinical practice are equivalent to those reported in the meta-analysis of randomised controlled trials conducted by Barrionuevo et al. [13]. In the real world, treatment effects may differ from those observed under controlled trial conditions, and future real-world evidence may provide further insights that could help improve the predictive power of the model.

In conclusion, our cross-sectional model highlights the substantial future clinical and economic burden of osteoporosis and osteoporotic fractures in postmenopausal women aged 70 years and over. This model also demonstrates that interventions aimed at improving osteoporosis care, specifically fracture risk assessment and adherence to anti-osteoporosis medications, would help to relieve this burden.

### Supplementary Information

Below is the link to the electronic supplementary material.

Supplementary file1 (DOCX 169 KB)

## Data Availability

Qualified researchers may request data from Amgen clinical studies. Complete details are available at https://wwwext.amgen.com/science/clinical-trials/clinical-data-transparency-practices.
