# Supplementary material for: Estimating the future clinical and economic benefits of improving osteoporosis diagnosis and treatment among postmenopausal women across eight European countries
Source: Arch Osteoporos. 2023 May 16;18(1):68. doi: 10.1007/s11657-023-01230-0 (PMC10188417; doi:10.1007/s11657-023-01230-0)
Supplement: Supplementary file 1 — Supplementary file1 (DOCX 169 KB) [file 11657_2023_1230_MOESM1_ESM.docx]

# Supplementary Material

## Supplementary Methods

### **Cohort model and its validation**

The cohort model used in this analysis was based on a previously published microsimulation model [1]. The microsimulation model was time consuming to run, so the study team adapted it into a cohort model, which reduced unwanted noise and reduced the run-time by more than 95%.

To ensure the validity of this adaptation, the results from the cohort model were compared with those from the microsimulation model. The estimated fracture counts differed by less than 0.1% between the microsimulation and cohort models. Face validity of the model structure, data sources and assumptions were evaluated by health economists, clinicians and country experts. Internal validity, in terms of accuracy of coding, was assessed by two additional reviewers and model results were evaluated against those of similar models built for other countries.

### **Estimation of untreated fracture risk**

*FRAX inputs*

The FRAX tool (paper charts version) was used to estimate the 10-year probability of hip or major osteoporotic fracture risk for an individual, based on the presence or absence of six clinical risk factors for that patient and their T-score or body mass index (BMI) [1]. The clinical risk factors are 1) smoking, 2) rheumatoid arthritis, 3) long-term glucocorticoid use, 4) excessive alcohol use, 5) parental history of a hip fracture and 6) previous fracture.

To estimate the proportion of the population in each FRAX paper chart for a given year, independence was assumed between age, BMI and clinical risk factors. It was also assumed that the BMI range for each country was normally distributed with mean and standard deviation parameters from McCloskey et al. [2]. Six true or false values were assigned, corresponding to each of the six clinical risk factors, and the number of true values counted. This assumed the expected proportion of people with a specific number of risk factors followed a Poisson binomial distribution [3]. Untreated fracture risk was estimated from FRAX paper charts based on population demographics and clinical risk fractures.

The extrapolation of hip fracture rates from FRAX to estimate vertebral and non-hip, non-vertebral rates using the country-specific distribution for each fracture type may be considered a limitation of this analysis. However, while alternative data sources, such as the FRAX simple chart using major osteoporotic fracture rates, were considered, these sources exclude certain fractures and hence methods to extrapolate rates to total fractures are lacking.

### **Estimation of treated fracture risk**

The treated fracture risk was estimated by adjusting the untreated fracture risk for the combination of anti-osteoporosis medications, corresponding treatment efficacy and medication adherence. For each country, these data were applied to the proportion of the population treated using a relative risk weighted by the treatments reported by McCloskey et al. [2]. The following treatments were included: oral bisphosphonate (which was assumed to be alendronate), denosumab, zoledronate, teriparatide and raloxifene. Treatment efficacy was based on a network meta‐analysis of clinical trials that examined the comparative effectiveness of various available pharmacological osteoporosis therapies [4]. To estimate the proportion of patients effectively treated, fracture rates were further adjusted for non-adherence. It was assumed that patients with a proportion of days covered greater than 80% were effectively treated, and that 40% of the treated population had a proportion of medication days covered greater than 80%. For treated individuals defined as non-adherent, the fracture risk was obtained by adding to the risk of the treated population [8]. The increased risk for non-adherent individuals was assumed to be the same across all countries.

# Supplementary Tables

**Supplementary Table S1** Data sources for model inputs

| Country | Distribution of fractures, % | Efficacy versus placebo (RR)  weighted based on treatment mix | Fracture cost, € | Anti-osteoporosis medication cost,^a^ € | BMD measurement, € | Status quo treatment rate among women ≥70, % | Identification rate of those at risk among those assessed, % | Treatment rate among those at risk, % |
| --- | --- | --- | --- | --- | --- | --- | --- | --- |
| Belgium | Hernlund et al. [3] | Barrionuevo et al. [4] | Hernlund et al. [3] 2019 € costs | Estimated using McCloskey et al. [2] treatment data and 2019 market costs | <https://ondpanon.riziv.fgov.be/Nomen/nl/455895/fees> | McCloskey et al. [2] (20170129 study data) | McCloskey et al. [2] (20170129 study data) | McCloskey et al. [2] (20170129 study data) |
| France | Hernlund et al. [3] | Barrionuevo et al. [4] | Fardellone et al. [5] 2019 € costs | Estimated using McCloskey et al. [2] treatment data and 2018 assurance maladie costs | CCAM ([https://www.ameli.fr/accueil−de−la−ccam/trouver−un−acte/fiche−abregee.php?code=PAQK007](https://www.ameli.fr/accueil-de-la-ccam/trouver-un-acte/fiche-abregee.php?code=PAQK007)) | McCloskey et al. [2] (20170129 study data) | McCloskey et al. [2] (20170129 study data) | McCloskey et al. [2] (20170129 study data) |
| Germany | Hernlund et al. [3] | Barrionuevo et al. [4] | Hernlund et al. [3] 2019 € costs | Estimated using McCloskey et al. [2] treatment data and market costs | Kanis et al. [6] | McCloskey et al. [2] (20170129 study data) | McCloskey et al. [2] (20170129 study data) | McCloskey et al. [2] (20170129 study data) |
| Ireland | Hernlund et al. [3] | Barrionuevo et al. [4] | Hernlund et al. [3] 2019 € costs | Estimated using McCloskey et al. [2] treatment data and market costs | Kanis et al. [6] | McCloskey et al. [2] (20170129 study data) | McCloskey et al. [2] (20170129 study data) | McCloskey et al. [2] (20170129 study data) |
| Poland | Hernlund et al. [3] | Barrionuevo et al. [4] | Hernlund et al. [3] 2019 € costs | Estimated using McCloskey et al. [2] treatment data and Poland National Health Fund costs | Kanis et al. [6] | McCloskey et al. [2] (20170129 study data) | McCloskey et al. [2] (20170129 study data) | McCloskey et al. [2] (20170129 study data) |
| Slovakia | Hernlund et al. [3] | Barrionuevo et al. [4] | 2016 € Slovakia costs <http://kategorizacia.mzsr.sk/Lieky/Download/RequestAttachment/36641> | Estimated using McCloskey et al. [2] treatment data and market costs | Kanis et al. [6] | McCloskey et al. [2] (20170129 study data) | McCloskey et al. [2] (20170129 study data) | McCloskey et al. [2] (20170129 study data) |
| Switzerland | Lippuner et al. [7] | Barrionuevo et al. [4] | Lippuner et al. [7] | Estimated using McCloskey et al. [2] treatment data and market costs | [https://www.kantonsspitalbaden.ch/Fachbereiche/Radiologie/Knochendichtemessung−(DEXA)/index.html](https://www.kantonsspitalbaden.ch/Fachbereiche/Radiologie/Knochendichtemessung-(DEXA)/index.html)  TARMED  [https://www.osteoporose−vorsorge.ch/diagnose−osteoporose.html](https://www.osteoporose-vorsorge.ch/diagnose-osteoporose.html) | McCloskey et al. [2] (20170129 study data) | McCloskey et al. [2] (20170129 study data) | McCloskey et al. [2] (20170129 study data) |
| UK | Borgström et al. [8] | Barrionuevo et al. [4] | TA464 Assessment Group Report | Estimated using McCloskey et al. [2] treatment data and market costs | NHS reference costs 2018/2019 | McCloskey et al. [2] (20170129 study data) | McCloskey et al. [2] (20170129 study data) | McCloskey et al. [2] (20170129 study data) |

^a^The anti-osteoporosis treatments included were based on the treatments utilised within the study conducted by McCloskey et al*.* The therapies included oral bisphosphonate (which was assumed to be alendronate), denosumab, zoledronate, teriparatide and raloxifene

*BMD*, bone mineral density; *CCAM*, Classification Commune Des Actes Medicaux; *RR*, relative risk

**Supplementary Table S2** Population demographics and clinical risk factors by country under the status quo

| Country | Total females ≥70 years old in 2020 | Femoral neck T-score,  mean (SD) | Body mass index,  mean (SD) | Rheumatoid arthritis,  (%) | Smoker, (%) | Excessive alcohol,  (%) | Glucocorticoid therapy, (%) | Personal history of fracture, (%) | Parental history of hip fracture,  % |
| --- | --- | --- | --- | --- | --- | --- | --- | --- | --- |
| Belgium | 909,965 | −1.7 (1.0) | 27.5 (5.1) | 3.6 | 6.7 | 1.2 | 3.6 | 31.5 | 10.5 |
| France | 5,755,944 | −1.3 (1.0) | 26.6 (5.3) | 5.0 | 5.0 | 0.6 | 3.1 | 27.3 | 13.8 |
| Germany | 7,617,401 | −1.4 (1.1) | 26.8 (4.7) | 4.9 | 6.9 | 0.8 | 4.5 | 29.8 | 8.3 |
| Ireland | 266,580 | −1.3 (1.2) | 27.6 (5.1) | 3.2 | 6.6 | 1.8 | 6.6 | 31.0 | 7.0 |
| Poland | 2,808,079 | −2.0 (1.0) | 28.2 (4.8) | 3.2 | 5.5 | 0.0 | 4.2 | 30.9 | 8.1 |
| Slovakia | 362,914 | −1.3 (1.0) | 29.3 (5.2) | 3.9 | 4.7 | 0.4 | 1.7 | 33.3 | 8.2 |
| Switzerland | 665,515 | −1.9 (1.1) | 26.3 (5.3) | 3.4 | 6.8 | 3.4 | 6.8 | 46.8 | 13.2 |
| UK | 5,082,343 | −1.2 (1.2) | 27.4 (5.4) | 4.2 | 7.0 | 3.8 | 8.2 | 31.4 | 9.8 |

Data sources: Total number of females ≥70 years obtained from Eurostat prediction model ([https://ec.europa.eu/eurostat/web/population−demography−migration−projections/population−projections−/database](https://ec.europa.eu/eurostat/web/population-demography-migration-projections/population-projections-/database)) for all countries except the UK, where data were obtained from the Office of National Statistics (<https://www.ons.gov.uk/peoplepopulationandcommunity/populationandmigration/populationprojections#datasets>) [9, 10]; clinical risk factor data obtained from McCloskey et al. [2] and associated 20170129 observational research study report (data on file, available upon request)

*SD*, standard deviation

**Supplementary Table S3** Treatment Efficacy (RR) for all countries

| **Treatment** | Hip fracture | Vertebral fracture | Non-hip non-vertebral fracture | Source |
| --- | --- | --- | --- | --- |
| Denosumab | 0.56 | 0.32 | 0.8 | Barrionuevo et al 2020. NHNV based on NV and includes hip. |
| Oral bisphosphonates |  |  |  |  |
| Alendronate | 0.61 | 0.57 | 0.84 | Barrionuevo et al 2020. NHNV based on NV and includes hip. |
| Oral ibandronate | 0.62 | 0.67 | 1.0 | Barrionuevo et al 2020. NHNV based on NV and includes hip. ibandorate RR >1. Assume no effect and equal to 1  ibandorate oral and IV values not differentiated |
| Oral risendronate | 0.73 | 0.61 | 0.78 | Barrionuevo et al 2020. NHNV based on NV and includes hip |
| Parenteral bisphosphonates |  |  |  |  |
| Ibandronate IV | 0.62 | 0.67 | 1.0 | Barrionuevo et al 2020. NHNV based on NV and includes hip ibandorate RR >1. Assume no effect and equal to 1  ibandorate oral and IV values not differentiated |
| Zoledronic acid | 0.6 | 0.38 | 0.79 | Barrionuevo et al 2020. NHNV based on NV and includes hip |
| PTH |  |  |  |  |
| Abaloparatide | - | - | - |  |
| Teriparatide | 0.64 | 0.27 | 0.62 | Barrionuevo et al 2020. NHNV based on NV and includes hip |
| SERM |  |  |  |  |
| Conjugated estrogens/bazedoxifene | 0.93 | 0.61 | 0.9 | Barrionuevo et al 2020. NHNV based on NV and includes hip |
| Raloxifene | 0.91 | 0.59 | 0.94 | Barrionuevo et al 2020. NHNV based on NV and includes hip |

*IV*, intravenous; *NHNV*, Non-Hip Non-Vertebral Fracture; *PTH*, parathyroid hormone; *RR*, relative risk; *SERM*, selective estrogen receptor modulators.

**Supplementary Table S4** Hypothetical interventions in main analysis and scenario analyses

|  |  | Rates improved from the status quo scenario | |
| --- | --- | --- | --- |
|  |  | Risk assessment | Medication non−adherence |
| Status quo |  | No change | No change |
| Main analysis | Intervention 1_50_ | 50% | No change |
|  | Intervention 2_50_ | No change | 50%^a^ |
|  | Intervention 3_50_ | 50% | 50% ^a^ |
| Scenario analysis | Intervention 1_10_ | 10% | No change |
|  | Intervention 2_10_ | No change | 10% ^a^ |
|  | Intervention 3_10_ | 10% | 10% ^a^ |
|  | Intervention 1_100_ | 100% | No change |
|  | Intervention 2_100_ | No change | 100% ^a^ |
|  | Intervention 3_100_ | 100% | 100% ^a^ |

a. Improvement on the percentages of non-adherence portion. For adherence rate of 40%, 50% improvement of 60% (the non-adherence part) results in +30% from 40% to 70%

**Supplementary Table S5** Risk assessment rates, treatment rates and adherence rates in the main analysis

|  | Risk assessment rate^a^ | | Treatment rate | | Adherence rate | |
| --- | --- | --- | --- | --- | --- | --- |
|  | Status quo | 50% improvement | Status quo | 50% improvement**^b^** | Status quo | 50% improvement^c^ |
| Belgium | 25% | 37.5% | 21.8% | 24.1% | 40% | 70% |
| France | 25% | 37.5% | 13.8% | 15.4% | 40% | 70% |
| Germany | 25% | 37.5% | 6.3% | 7.0% | 40% | 70% |
| Ireland | 25% | 37.5% | 28.6% | 31.4% | 40% | 70% |
| Poland | 25% | 37.5% | 7.7% | 8.5% | 40% | 70% |
| Slovakia | 25% | 37.5% | 12.9% | 14.2% | 40% | 70% |
| Switzerland | 25% | 37.5% | 27.3% | 30.7% | 40% | 70% |
| UK | 25% | 37.5% | 20.4% | 22.5% | 40% | 70% |

^a^McCloskey et al. [2] reported that 30% (*n* = 641/2077) of women at increased risk of fracture had an osteoporosis diagnosis; it was assumed for this model that 25% of women aged 70 years or older were assessed for fracture risk

^b^ New treatment rates were calculated based on information listed in Supplementary Table S1

**^c^** 50% improvement of 60% (the non-adherence part) results in +30% from 40% to 70%

**Supplementary Table S6** Fracture and cost reduction^a^ by hypothetical intervention and country

| **Country** | **Intervention** |  | **2020** | **2025** | **2030** | **2035** | **2040** |
| --- | --- | --- | --- | --- | --- | --- | --- |
| **Belgium** |  | | | | | | |
|  | **Intervention 1_10_ (10% improvement)** | Fractures | −5223 | −2894 | −3391 | −6737 | −6061 |
|  |  | n/100,000 population | −574 | −295 | −314 | −567 | −476 |
|  |  |  | −10.7% | −5.6% | −6.0% | −10.6% | −8.6% |
|  |  | Costs, in millions | −€ 26.5 | −€ 14.5 | −€ 17.1 | −€ 34.2 | −€ 30.7 |
|  |  | n/100,000 population | −€ 2.9 | −€ 1.5 | −€ 1.6 | −€ 2.9 | −€ 2.4 |
|  |  |  | −7.7% | −4.0% | −4.3% | −7.6% | −6.2% |
|  | **Intervention 2_10_ (10% improvement)** | Fractures | −2551 | −3223 | −3780 | −3287 | −3652 |
|  |  | n/100,000 population | −280 | −328 | −350 | −276 | −287 |
|  |  |  | −5.2% | −6.3% | −6.7% | −5.2% | −5.2% |
|  |  | Costs, in millions | −€ 10.9 | −€ 16.0 | −€ 19.1 | −€ 14.1 | −€ 15.6 |
|  |  | n/100,000 population | −€ 1.2 | −€ 1.6 | −€ 1.8 | −€ 1.2 | −€ 1.2 |
|  |  |  | −3.2% | −4.4% | −4.8% | −3.1% | −3.2% |
|  | **Intervention 3_10_ (10% improvement)** | Fractures | −6528 | −4357 | −4908 | −8523 | −7007 |
|  |  | n/100,000 population | −717 | −444 | −455 | −717 | −551 |
|  |  |  | −13.4% | −8.5% | −8.7% | −13.4% | −10.0% |
|  |  | Costs, in millions | −€ 33.5 | −€ 22.4 | −€ 25.3 | −€ 43.8 | −€ 35.9 |
|  |  | n/100,000 population | −€ 3.7 | −€ 2.3 | −€ 2.3 | −€ 3.7 | −€ 2.8 |
|  |  |  | −9.8% | −6.1% | −6.3% | −9.8% | −7.3% |
|  | **Intervention 1_50_ (50% improvement)** | Fractures | −6678 | −5197 | −5787 | −8562 | −7491 |
|  |  | N/100,000 population | −734 | −529 | −536 | −720 | −589 |
|  |  |  | −13.7% | −10.1% | −10.2% | −13.4% | −10.7% |
|  |  | Costs, in millions | −€ 31.0 | −€ 23.1 | −€ 25.8 | −€ 39.7 | −€ 33.7 |
|  |  | N/100,000 population | −€ 3.4 | −€ 2.3 | −€ 2.4 | −€ 3.3 | −€ 2.6 |
|  |  |  | −9.0% | −6.3% | −6.4% | −8.9% | −6.8% |
|  | **Intervention 2_50_ (50% improvement)** | Fractures | −7908 | −8111 | −9013 | −10192 | −9696 |
|  |  | N/100,000 population | −869 | −826 | −835 | −857 | −762 |
|  |  |  | −16.2% | −15.8% | −15.9% | −16.0% | −13.8% |
|  |  | Costs, in millions | −€ 39.1 | −€ 41.7 | −€ 46.5 | −€ 50.4 | −€ 47.4 |
|  |  | N/100,000 population | −€ 4.3 | −€ 4.2 | −€ 4.3 | −€ 4.2 | −€ 3.7 |
|  |  |  | −11.4% | −11.4% | −11.6% | −11.2% | −9.6% |
|  | **Intervention 3_50_ (50% improvement)** | Fractures | −12565 | −12964 | −13954 | −16344 | −16266 |
|  |  | N/100,000 population | −1381 | −1321 | −1293 | −1374 | −1278 |
|  |  |  | −25.8% | −25.2% | −24.7% | −25.7% | −23.2% |
|  |  | Costs, in millions | −€ 61.8 | −€ 63.3 | −€ 68.2 | −€ 80.3 | −€ 79.9 |
|  |  | N/100,000 population | −€ 6.8 | −€ 6.4 | −€ 6.3 | −€ 6.8 | −€ 6.3 |
|  |  |  | −18.0% | −17.3% | −17.0% | −17.9% | −16.2% |
|  | **Intervention 1_100_ (100% improvement)** | Fractures | −9409 | −8268 | −9116 | −12470 | −10679 |
|  |  | N/100,000 population | −1034 | −842 | −845 | −1049 | −839 |
|  |  |  | −19.3% | −16.1% | −16.1% | −19.6% | −15.2% |
|  |  | Costs, in millions | −€ 41.0 | −€ 34.6 | −€ 38.3 | −€ 54.5 | −€ 44.7 |
|  |  | N/100,000 population | −€ 4.5 | −€ 3.5 | −€ 3.6 | −€ 4.6 | −€ 3.5 |
|  |  |  | −12.0% | −9.5% | −9.6% | −12.2% | −9.1% |
|  | **Intervention 2_100_ (100% improvement)** | Fractures | −12080 | −13104 | −14522 | −15645 | −17487 |
|  |  | N/100,000 population | −1328 | −1335 | −1346 | −1316 | −1374 |
|  |  |  | −24.8% | −25.4% | −25.7% | −24.6% | −24.9% |
|  |  | Costs, in millions | −€ 60.1 | −€ 66.4 | −€ 73.8 | −€ 77.8 | −€ 86.9 |
|  |  | N/100,000 population | −€ 6.6 | −€ 6.8 | −€ 6.8 | −€ 6.5 | −€ 6.8 |
|  |  |  | −17.5% | −18.2% | −18.4% | −17.4% | −17.6% |
|  | **Intervention 3_100_ (100% improvement)** | Fractures | −16357 | −16883 | −18530 | −21373 | −21594 |
|  |  | N/100,000 population | −1797 | −1720 | −1717 | −1797 | −1697 |
|  |  |  | −33.6% | −32.8% | −32.7% | −33.6% | −30.8% |
|  |  | Costs, in millions | −€ 68.5 | −€ 72.2 | −€ 79.6 | −€ 89.3 | −€ 92.0 |
|  |  | N/100,000 population | −€ 7.5 | −€ 7.4 | −€ 7.4 | −€ 7.5 | −€ 7.2 |
|  |  |  | −20.0% | −19.8% | −19.8% | −19.9% | −18.6% |
| **France** |  | | | | | | |
|  | **Intervention 1_10_ (10% improvement)** | Fractures | −17814 | −20815 | −30993 | −39824 | −33081 |
|  |  | N/100,000 population | −309 | −324 | −438 | −516 | −401 |
|  |  |  | −5.9% | −6.3% | −8.4% | −9.5% | −7.2% |
|  |  | Costs, in millions | −€ 245.4 | −€ 286.8 | −€ 429.9 | −€ 554.4 | −€ 459.1 |
|  |  | N/100,000 population | −€ 4.3 | −€ 4.5 | −€ 6.1 | −€ 7.2 | −€ 5.6 |
|  |  |  | −5.3% | −5.7% | −7.6% | −8.7% | −6.5% |
|  | **Intervention 2_10_ (10% improvement)** | Fractures | −19863 | −10769 | −20666 | −25955 | −13890 |
|  |  | N/100,000 population | −345 | −168 | −292 | −336 | −168 |
|  |  |  | −6.6% | −3.3% | −5.6% | −6.2% | −3.0% |
|  |  | Costs, in millions | −€ 272.3 | −€ 143.1 | −€ 282.7 | −€ 351.0 | −€ 180.6 |
|  |  | N/100,000 population | −€ 4.7 | −€ 2.2 | −€ 4.0 | −€ 4.5 | −€ 2.2 |
|  |  |  | −5.9% | −2.8% | −5.0% | −5.5% | −2.6% |
|  | **Intervention 3_10_ (10% improvement)** | Fractures | −29493 | −21433 | −31612 | −56182 | −50970 |
|  |  | N/100,000 population | −512 | −334 | −447 | −728 | −618 |
|  |  |  | −9.7% | −6.5% | −8.5% | −13.4% | −11.0% |
|  |  | Costs, in millions | −€ 409.5 | −€ 294.9 | −€ 438.0 | −€ 784.3 | −€ 710.4 |
|  |  | N/100,000 population | −€ 7.1 | −€ 4.6 | −€ 6.2 | −€ 10.2 | −€ 8.6 |
|  |  |  | −8.8% | −5.9% | −7.7% | −12.3% | −10.1% |
|  | **Intervention 1_50_ (50% improvement)** | Fractures | −37174 | −29025 | −41832 | −58103 | −48069 |
|  |  | N/100,000 population | −646 | −452 | −592 | −753 | −583 |
|  |  |  | −12.3% | −8.8% | −11.3% | −13.8% | −10.4% |
|  |  | Costs, in millions | −€ 494.3 | −€ 375.8 | −€ 553.0 | −€ 780.8 | −€ 637.1 |
|  |  | N/100,000 population | −€ 8.6 | −€ 5.9 | −€ 7.8 | −€ 10.1 | −€ 7.7 |
|  |  |  | −10.7% | −7.5% | −9.8% | −12.2% | −9.0% |
|  | **Intervention 2_50_ (50% improvement)** | Fractures | −34313 | −34117 | −46337 | −47581 | −53975 |
|  |  | N/100,000 population | −596 | −532 | −655 | −616 | −654 |
|  |  |  | −11.3% | −10.4% | −12.5% | −11.3% | −11.7% |
|  |  | Costs, in millions | −€ 474.5 | −€ 469.4 | −€ 641.4 | −€ 653.6 | −€ 743.1 |
|  |  | N/100,000 population | −€ 8.2 | −€ 7.3 | −€ 9.1 | −€ 8.5 | −€ 9.0 |
|  |  |  | −10.3% | −9.3% | −11.3% | −10.2% | −10.6% |
|  | **Intervention 3_50_ (50% improvement)** | Fractures | −61876 | −57458 | −72713 | −88510 | −97506 |
|  |  | N/100,000 population | −1075 | −895 | −1028 | −1146 | −1182 |
|  |  |  | −20.4% | −17.5% | −19.6% | −21.1% | −21.1% |
|  |  | Costs, in millions | −€ 838.8 | −€ 772.5 | −€ 983.9 | −€ 1,205.0 | −€ 1,329.3 |
|  |  | N/100,000 population | −€ 14.6 | −€ 12.0 | −€ 13.9 | −€ 15.6 | −€ 16.1 |
|  |  |  | −18.1% | −15.4% | −17.4% | −18.8% | −18.9% |
|  | **Intervention 1_100_ (100% improvement)** | Fractures | −49719 | −41166 | −54698 | −69744 | −77163 |
|  |  | N/100,000 population | −864 | −641 | −774 | −903 | −936 |
|  |  |  | −16.4% | −12.5% | −14.8% | −16.6% | −16.7% |
|  |  | Costs, in millions | −€ 640.7 | −€ 513.4 | −€ 697.4 | −€ 904.8 | −€ 1,004.4 |
|  |  | N/100,000 population | −€ 11.1 | −€ 8.0 | −€ 9.9 | −€ 11.7 | −€ 12.2 |
|  |  |  | −13.8% | −10.2% | −12.3% | −14.1% | −14.3% |
|  | **Intervention 2_100_ (100% improvement)** | Fractures | −64501 | −59948 | −77375 | −78920 | −88265 |
|  |  | N/100,000 population | −1121 | −934 | −1094 | −1022 | −1070 |
|  |  |  | −21.3% | −18.2% | −20.9% | −18.8% | −19.1% |
|  |  | Costs, in millions | −€ 895.9 | −€ 830.2 | −€ 1,074.6 | −€ 1,093.6 | −€ 1,224.5 |
|  |  | N/100,000 population | −€ 15.6 | −€ 12.9 | −€ 15.2 | −€ 14.2 | −€ 14.8 |
|  |  |  | −19.4% | −16.5% | −19.0% | −17.1% | −17.4% |
|  | **Intervention 3_100_ (100% improvement)** | Fractures | −87168 | −82220 | −103,158 | −106,986 | −118,363 |
|  |  | N/100,000 population | −1514 | −1281 | −1459 | −1386 | −1435 |
|  |  |  | −28.8% | −25.0% | −27.9% | −25.5% | −25.6% |
|  |  | Costs, in millions | −€ 1,133.7 | −€ 1,056.3 | −€ 1,339.8 | −€ 1,384.5 | −€ 1,537.3 |
|  |  | N/100,000 population | −€ 19.7 | −€ 16.5 | −€ 18.9 | −€ 17.9 | −€ 18.6 |
|  |  |  | −24.5% | −21.0% | −23.7% | −21.6% | −21.8% |
| **Germany** |  | | | | | | |
|  | **Intervention 1_10_ (10% improvement)** | Fractures | −19791 | −23984 | −22160 | −11482 | −26337 |
|  |  | N/100,000 population | −260 | −300 | −259 | −122 | −257 |
|  |  |  | −4.8% | −5.6% | −5.0% | −2.4% | −4.9% |
|  |  | Costs, in millions | −€ 179.0 | −€ 217.8 | −€ 200.3 | −€ 99.4 | −€ 238.0 |
|  |  | N/100,000 population | −€ 2.3 | −€ 2.7 | −€ 2.3 | −€ 1.1 | −€ 2.3 |
|  |  |  | −4.0% | −4.6% | −4.1% | −1.9% | −4.0% |
|  | **Intervention 2_10_ (10% improvement)** | Fractures | −7215 | −6780 | −11202 | −12010 | −14062 |
|  |  | N/100,000 population | −95 | −85 | −131 | −127 | −137 |
|  |  |  | −1.8% | −1.6% | −2.5% | −2.5% | −2.6% |
|  |  | Costs, in millions | −€ 52.6 | −€ 42.8 | −€ 85.6 | −€ 110.5 | −€ 110.1 |
|  |  | N/100,000 population | −€ 0.7 | −€ 0.5 | −€ 1.0 | −€ 1.2 | −€ 1,1 |
|  |  |  | −1.2% | −0.9% | −1.8% | −2.1% | −1.9% |
|  | **Intervention 3_10_ (10% improvement)** | Fractures | −24401 | −24411 | −27936 | −16743 | −32901 |
|  |  | N/100,000 population | −320 | −305 | −327 | −177 | −320 |
|  |  |  | −6.0% | −5.7% | −6.2% | −3.5% | −6.1% |
|  |  | Costs, in millions | −€ 222.5 | −€ 221.9 | −€ 254.8 | −€ 149.1 | −€ 300.0 |
|  |  | N/100,000 population | −€ 2.9 | −€ 2.8 | −€ 3.0 | −€ 1.6 | −€ 2.9 |
|  |  |  | −5.0% | −4.7% | −5.2% | −2.8% | −5.1% |
|  | **Intervention 1_50_ (50% improvement)** | Fractures | −23061 | −23984 | −39037 | −24152 | −46352 |
|  |  | N/100,000 population | −303 | −300 | −457 | −256 | −451 |
|  |  |  | −5.6% | −5.6% | −8.7% | −5.0% | −8.6% |
|  |  | Costs, in millions | −€ 182.0 | −€ 189.0 | −€ 327.2 | −€ 183.8 | −€ 388.0 |
|  |  | N/100,000 population | −€ 2.4 | −€ 2.4 | −€ 3.8 | −€ 1.9 | −€ 3.8 |
|  |  |  | −4.1% | −4.0% | −6.7% | −3.5% | −6.6% |
|  | **Intervention 2_50_ (50% improvement)** | Fractures | −21389 | −21834 | −28842 | −31968 | −35620 |
|  |  | N/100,000 population | −281 | −273 | −338 | −339 | −347 |
|  |  |  | −5.2% | −5.1% | −6.4% | −6.6% | −6.6% |
|  |  | Costs, in millions | −€ 185.1 | −€ 183.6 | −€ 249.3 | −€ 295.7 | −€ 310.2 |
|  |  | N/100,000 population | −€ 2.4 | −€ 2.3 | −€ 2.9 | −€ 3.1 | −€ 3.0 |
|  |  |  | −4.1% | −3.9% | −5.1% | −5.6% | −5.3% |
|  | **Intervention 3_50_ (50% improvement)** | Fractures | −44503 | −45921 | −57569 | −58473 | −68292 |
|  |  | N/100,000 population | −584 | −574 | −674 | −619 | −665 |
|  |  |  | −10.9% | −10.7% | −12.9% | −12.1% | −12.6% |
|  |  | Costs, in millions | −€ 381.7 | −€ 393.5 | −€ 499.2 | −€ 500.5 | −€ 591.7 |
|  |  | N/100,000 population | −€ 5.0 | −€ 4.9 | −€ 5.8 | −€ 5.3 | −€ 5.8 |
|  |  |  | −8.5% | −8.4% | −10.2% | −9.4% | −10.0% |
|  | **Intervention 1_100_ (100% improvement)** | Fractures | −34170 | −39767 | −39037 | −52695 | −70911 |
|  |  | N/100,000 population | −449 | −497 | −457 | −558 | −691 |
|  |  |  | −8.3% | −9.3% | −8.7% | −10.9% | −13.1% |
|  |  | Costs, in millions | −€ 251.6 | −€ 300.6 | −€ 288.8 | −€ 405.2 | −€ 568.2 |
|  |  | N/100,000 population | −€ 3.3 | −€ 3.8 | −€ 3.4 | −€ 4.3 | −€ 5.5 |
|  |  |  | −5.6% | −6.4% | −5.9% | −7.6% | −9.6% |
|  | **Intervention 2_100_ (100% improvement)** | Fractures | −41645 | −46437 | −46259 | −48323 | −55191 |
|  |  | N/100,000 population | −547 | −580 | −542 | −512 | −538 |
|  |  |  | −10.2% | −10.8% | −10.3% | −10.0% | −10.2% |
|  |  | Costs, in millions | −€ 373.5 | −€ 412.8 | −€ 412.0 | −€ 445.9 | −€ 493.0 |
|  |  | N/100,000 population | −€ 4.9 | −€ 5.2 | −€ 4.8 | −€ 4.7 | −€ 4.8 |
|  |  |  | −8.4% | −8.8% | −8.4% | −8.4% | −8.4% |
|  | **Intervention 3_100_ (100% improvement)** | Fractures | −53305 | −58289 | −59495 | −82463 | −97020 |
|  |  | N/100,000 population | −700 | −728 | −697 | −873 | −945 |
|  |  |  | −13.0% | −13.6% | −13.3% | −17.0% | −17.9% |
|  |  | Costs, in millions | −€ 400.7 | −€ 438.6 | −€ 444.1 | −€ 634.2 | −€ 737.3 |
|  |  | N/100,000 population | −€ 5.3 | −€ 5.5 | −€ 5.2 | −€ 6.7 | −€ 7.2 |
|  |  |  | −9.0% | −9.4% | −9.1% | −12.0% | −12.5% |
| **Ireland** |  | | | | | | |
|  | **Intervention 1_10_ (10% improvement)** | Fractures | −738 | −880 | −1184 | −774 | −1938 |
|  |  | N/100,000 population | −277 | −279 | −319 | −180 | −398 |
|  |  |  | −5.0% | −5.0% | −5.7% | −3.1% | −6.8% |
|  |  | Costs, in millions | −€ 3.8 | −€ 4.6 | −€ 6.3 | −€ 3.8 | −€ 10.8 |
|  |  | N/100,000 population | −€ 1.4 | −€ 1.4 | −€ 1.7 | −€ 0.9 | −€ 2.2 |
|  |  |  | −2.9% | −2.9% | −3.4% | −1.7% | −4.3% |
|  | **Intervention 2_10_ (10% improvement)** | Fractures | −970 | −1204 | −950 | −1188 | −1763 |
|  |  | N/100,000 population | −364 | −381 | −256 | −276 | −362 |
|  |  |  | −6.6% | −6.9% | −4.5% | −4.8% | −6.2% |
|  |  | Costs, in millions | −€ 5.4 | −€ 6,8 | −€ 5.5 | −€ 6.8 | −€ 9.6 |
|  |  | N/100,000 population | −€ 2.0 | −€ 2.1 | −€ 1.5 | −€ 1.6 | −€ 2.0 |
|  |  |  | −4.1% | −4.4% | −2.9% | −3.1% | −3.8% |
|  | **Intervention 3_10_ (10% improvement)** | Fractures | −1313 | −1616 | −1858 | −1647 | −3328 |
|  |  | N/100,000 population | −493 | −511 | −501 | −383 | −683 |
|  |  |  | −8.9% | −9.2% | −8.9% | −6.7% | −11.7% |
|  |  | Costs, in millions | −€ 7.4 | −€ 9.1 | −€ 10.5 | −€ 9.2 | −€ 19.5 |
|  |  | N/100,000 population | −€ 2.8 | −€ 2.9 | −€ 2.8 | −€ 2.1 | −€ 4.0 |
|  |  |  | −5.6% | −5.9% | −5.7% | −4.2% | −7.8% |
|  | **Intervention 1_50_ (50% improvement)** | Fractures | −1685 | −1285 | −2083 | −2263 | −3118 |
|  |  | N/100,000 population | −632 | −406 | −562 | −526 | −640 |
|  |  |  | −11.4% | −7.4% | −9.9% | −9.2% | −10.9% |
|  |  | Costs, in millions | −€ 6.5 | −€ 3.8 | −€ 8.1 | −€ 8.6 | −€ 13.1 |
|  |  | N/100,000 population | −€ 2.4 | −€ 1.2 | −€ 2.2 | −€ 2.0 | −€ 2.7 |
|  |  |  | −4.9% | −2.4% | −4.4% | −3.9% | −5.2% |
|  | **Intervention 2_50_ (50% improvement)** | Fractures | −2172 | −2412 | −2977 | −3496 | −4388 |
|  |  | N/100,000 population | −815 | −763 | −803 | −813 | −900 |
|  |  |  | −14.7% | −13.8% | −14.2% | −14.2% | −15.4% |
|  |  | Costs, in millions | −€ 14.4 | −€ 14.1 | −€ 17.8 | −€ 20.9 | −€ 25.6 |
|  |  | N/100,000 population | −€ 5.4 | −€ 4.5 | −€ 4.8 | −€ 4.9 | −€ 5.3 |
|  |  |  | −10.9% | −9.1% | −9.6% | −9.6% | −10.2% |
|  | **Intervention 3_50_ (50% improvement)** | Fractures | −3873 | −3723 | −4988 | −5646 | −7163 |
|  |  | N/100,000 population | −1453 | −1178 | −1345 | −1312 | −1470 |
|  |  |  | −26.2% | −21.3% | −23.8% | −22.9% | −25.1% |
|  |  | Costs, in millions | −€ 21.5 | −€ 18.6 | −€ 25.7 | −€ 29.1 | −€ 37.9 |
|  |  | N/100,000 population | −€ 8.1 | −€ 5.9 | −€ 6.9 | −€ 6.8 | −€ 7.8 |
|  |  |  | −16.2% | −12.0% | −13.9% | −13.3% | −15.2% |
|  | **Intervention 1_100_ (100% improvement)** | Fractures | 2243 | 2756 | 23992 | 3848 | 4727 |
|  |  | N/100,000 population | −841 | −872 | −1077 | −894 | −970 |
|  |  |  | −15.2% | −15.8% | −19.1% | −15.6% | −16.6% |
|  |  | Costs, in millions | −€ 6.7 | −€ 8.5 | −€ 14.6 | −€ 12.2 | −€ 16.8 |
|  |  | N/100,000 population | −€ 2.5 | −€ 2.7 | −€ 3.9 | −€ 2.8 | −€ 3.5 |
|  |  |  | −5.0% | −5.4% | −7.9% | −5.6% | −6.7% |
|  | **Intervention 2_100_ (100% improvement)** | Fractures | −4021 | −4631 | −5453 | −6480 | −7450 |
|  |  | N/100,000 population | −1508 | −1465 | −1471 | −1506 | −1529 |
|  |  |  | −27.2% | −26.5% | −26.0% | −26.3% | −26.1% |
|  |  | Costs, in millions | −€ 25.3 | −€ 27.1 | −€ 32.5 | −€ 38.6 | −€ 44.0 |
|  |  | N/100,000 population | −€ 9.5 | −€ 8.6 | −€ 8.8 | −€ 9.0 | −€ 9.0 |
|  |  |  | −19.1% | −17.5% | −17.6% | −17.7% | −17.6% |
|  | **Intervention 3_100_ (100% improvement)** | Fractures | −5,029 | −5,870 | −7,457 | −8,816 | −9,529 |
|  |  | N/100,000 population | −1887 | −1857 | −2011 | −2049 | −1955 |
|  |  |  | −34.1% | −33.6% | −35.6% | −35.8% | −33.4% |
|  |  | Costs, in millions | −€ 22.5 | −€ 24.0 | −€ 31.2 | −€ 37.1 | −€ 41.0 |
|  |  | N/100,000 population | −€ 8.4 | −€ 7.6 | −€ 8.4 | −€ 8.6 | −€ 8.4 |
|  |  |  | −17.0% | −15.5% | −16.8% | −17.0% | −16.4% |
| **Poland** |  | | | | | | |
|  | **Intervention 1_10_ (10% improvement)** | Fractures | −5742 | −4487 | −5941 | −8947 | −9133 |
|  |  | N/100,000 population | −204 | −135 | −156 | −222 | −224 |
|  |  |  | −6.7% | −4.7% | −5.3% | −7.0% | −6.5% |
|  |  | Costs, in millions | −€ 10.7 | −€ 8.1 | −€ 10.8 | −€ 16.8 | −€ 17.4 |
|  |  | N/100,000 population | −€ 0.4 | −€ 0.2 | −€ 0.3 | −€ 0.4 | −€ 0.4 |
|  |  |  | −4.6% | −3.1% | −3.5% | −4.8% | −4.6% |
|  | **Intervention 2_10_ (10% improvement)** | Fractures | −3971 | −2037 | −2844 | −5612 | −4829 |
|  |  | N/100,000 population | −141 | −61 | −75 | −139 | −118 |
|  |  |  | −4.6% | −2.1% | −2.6% | −4.4% | −3.4% |
|  |  | Costs, in millions | −€ 6.2 | −€ 2.5 | −€ 3.3 | −€ 8.3 | −€ 7.5 |
|  |  | N/100,000 population | −€ 0.2 | −€ 0.07 | −€ 0.09 | −€ 0.2 | −€ 0.2 |
|  |  |  | −2.7% | −0.9% | −1.1% | −2.4% | −1.9% |
|  | **Intervention 3_10_ (10% improvement)** | Fractures | −7221 | −5546 | −8225 | −10,968 | −9,330 |
|  |  | N/100,000 population | −257 | −167 | −216 | −272 | −229 |
|  |  |  | −8.4% | −5.8% | −7.4% | −8.5% | −6.6% |
|  |  | Costs, in millions | −€ 13.8 | −€ 10.2 | −€ 15.4 | −€ 20.9 | −€ 17.9 |
|  |  | N/100,000 population | −€ 0.5 | −€ 0.3 | −€ 0.4 | −€ 0.5 | −€ 0.4 |
|  |  |  | −5.9% | −3.9% | −5.0% | −5.9% | −4.7% |
|  | **Intervention 1_50_ (50% improvement)** | Fractures | −7,115 | −8,536 | −10,148 | −8,947 | −9,269 |
|  |  | N/100,000 population | −253 | −258 | −266 | −222 | −227 |
|  |  |  | −8.3% | −8.9% | −9.1% | −7.0% | −6.6% |
|  |  | Costs, in millions | −€ 10.7 | −€ 12.6 | −€ 15.2 | −€ 12.8 | −€ 13.6 |
|  |  | N/100,000 population | −€ 0.4 | −€ 0.4 | −€ 0.4 | −€ 0.3 | −€ 0.3 |
|  |  |  | −4.6% | −4.8% | −5.0% | −3.6% | −3.6% |
|  | **Intervention 2_50_ (50% improvement)** | Fractures | −7674 | −8520 | −9744 | −10,367 | −10,245 |
|  |  | N/100,000 population | −273 | −257 | −256 | −258 | −251 |
|  |  |  | −9.0% | −8.9% | −8.7% | −8.1% | −7.3% |
|  |  | Costs, in millions | −€ 13.5 | −€ 15.0 | −€ 16.6 | −€ 17.8 | −€ 18.4 |
|  |  | N/100,000 population | −€ 0.5 | −€ 0.5 | −€ 0.4 | −€ 0.4 | −€ 0.5 |
|  |  |  | −5.8% | −5.7% | −5.4% | −5.0% | −4.8% |
|  | **Intervention 3_50_ (50% improvement)** | Fractures | −13,204 | −15,472 | −17,597 | −17,165 | −16,916 |
|  |  | N/100,000 population | −470 | −467 | −462 | −426 | −415 |
|  |  |  | −15.4% | −16.1% | −15.8% | −13.4% | −12.0% |
|  |  | Costs, in millions | −€ 22.6 | −€ 26.1 | −€ 29.6 | −€ 29.1 | −€ 29.0 |
|  |  | N/100,000 population | −€ 0.8 | −€ 0.8 | −€ 0.8 | −€ 0.7 | −€ 0.7 |
|  |  |  | −9.6% | −9.9% | −9.7% | −8.3% | −7.6% |
|  | **Intervention 1_100_ (100% improvement)** | Fractures | −11,027 | −9,527 | −11,905 | −10,815 | −15,861 |
|  |  | N/100,000 population | −393 | −288 | −312 | −269 | −389 |
|  |  |  | −12.9% | −9.9% | −10.7% | −8.4% | −11.3% |
|  |  | Costs, in millions | −€ 14.8 | −€ 10.5 | −€ 14.0 | −€ 11.5 | −€ 21.4 |
|  |  | N/100,000 population | −€ 0.5 | −€ 0.3 | −€ 0.4 | −€ 0.3 | −€ 0.5 |
|  |  |  | −6.3% | −4.0% | −4.6% | −3.3% | −5.6% |
|  | **Intervention 2_100_ (100% improvement)** | Fractures | −13,872 | −12,358 | −15,091 | −21,036 | −18,736 |
|  |  | N/100,000 population | −494 | −373 | −396 | −523 | −460 |
|  |  |  | −16.2% | −12.9% | −13.5% | −16.4% | −13.3% |
|  |  | Costs, in millions | −€ 25.4 | −€ 22.4 | −€ 26.9 | −€ 38.2 | −€ 35.1 |
|  |  | N/100,000 population | −€ 0.9 | −€ 0.7 | −€ 0.7 | −€ 0.9 | −€ 0.9 |
|  |  |  | −10.8% | −8.5% | −8.8% | −10.9% | −9.2% |
|  | **Intervention 3_100_ (100% improvement)** | Fractures | −18,055 | −16,640 | −19,125 | −21,258 | −26,010 |
|  |  | N/100,000 population | −643 | −502 | −502 | −528 | −638 |
|  |  |  | −21.1% | −17.4% | −17.2% | −16.5% | −18.5% |
|  |  | Costs, in millions | −€ 24.2 | −€ 20.0 | −€ 23.0 | −€ 28.6 | −€ 35.1 |
|  |  | N/100,000 population | −€ 0.9 | −€ 0.6 | −€ 0.6 | −€ 0.7 | −€ 0.9 |
|  |  |  | −10.3% | −7.6% | −7.5% | −8.1% | −9.2% |
| **Slovakia** |  | | | | | | |
|  | **Intervention 1_10_ (10% improvement)** | Fractures | −1365 | −755 | −1225 | −1986 | −2105 |
|  |  | N/100,000 population | −376 | −175 | −247 | −370 | −377 |
|  |  |  | −8.1% | −3.9% | −5.3% | −7.6% | −7.5% |
|  |  | Costs, in millions | −€ 1.6 | −€ 0.7 | −€ 1.3 | −€ 2.4 | −€ 2.6 |
|  |  | N/100,000 population | −€ 0.4 | −€ 0.2 | −€ 0.3 | −€ 0.5 | −€ 0.4 |
|  |  |  | −3.4% | −1.3% | −2.0% | −3.3% | −3.3% |
|  | **Intervention 2_10_ (10% improvement)** | Fractures | −348 | −439 | −475 | −938 | −988 |
|  |  | N/100,000 population | −96 | −102 | −96 | −175 | −177 |
|  |  |  | −2.1% | −2.2% | −2.0% | −3.6% | −3.5% |
|  |  | Costs, in millions | € 0.2 | −€ 0.2 | € 0.4 | −€ 0.08 | −€ 0.1 |
|  |  | N/100,000 population | € 0.06 | −€ 0.04 | € 0.7 | −€ 0.1 | −€ 0.02 |
|  |  |  | 0.5% | −0.3% | 0.6% | −0.1% | −0.1% |
|  | **Intervention 3_10_ (10% improvement)** | Fractures | −1729 | −947 | −1600 | −2433 | −2512 |
|  |  | N/100,000 population | −476 | −219 | −323 | −453 | −450 |
|  |  |  | −10.2% | −4.8% | −6.9% | −9.3% | −9.0% |
|  |  | Costs, in millions | −€ 2.2 | −€ 1.0 | −€ 1.9 | −€ 3.1 | −€ 3.3 |
|  |  | N/100,000 population | −€ 0.6 | −€ 0.2 | −€ 0.4 | −€ 0.6 | −€ 0.6 |
|  |  |  | −4.5% | −1.8% | −2.9% | −4.3% | −4.2% |
|  | **Intervention 1_50_ (50% improvement)** | Fractures | −1583 | −972 | −2191 | −2243 | −2212 |
|  |  | N/100,000 population | −436 | −225 | −442 | −418 | −396 |
|  |  |  | −9.4% | −5.0% | −9.5% | −8.5% | −7.9% |
|  |  | Costs, in millions | −€ 0.8 | € 0.4 | −€ 1.1 | −€ 1.1 | −€ 1.0 |
|  |  | N/100,000 population | −€ 0.2 | € 0.09 | −€ 0.2 | −€ 0.2 | −€ 0.2 |
|  |  |  | −1.7% | 0.7% | −1.7% | −1.5% | −1.3% |
|  | **Intervention 2_50_ (50% improvement)** | Fractures | −1381 | −1500 | −1793 | −2548 | −2743 |
|  |  | N/100,000 population | −380 | −347 | −362 | −474 | −491 |
|  |  |  | −8.2% | −7.7% | −7.7% | −9.7% | −9.8% |
|  |  | Costs, in millions | −€ 1.2 | −€ 1.6 | −€ 1.4 | −€ 2.3 | −€ 2.6 |
|  |  | N/100,000 population | −€ 0.3 | −€ 0.4 | −€ 0.3 | −€ 0.4 | −€ 0.5 |
|  |  |  | −2.4% | −2.8% | −2.2% | −3.2% | −3.3% |
|  | **Intervention 3_50_ (50% improvement)** | Fractures | −2928 | −2636 | −3937 | −4411 | −4469 |
|  |  | N/100,000 population | −807 | −609 | −795 | −821 | −800 |
|  |  |  | −17.4% | −13.5% | −17.0% | −16.8% | −15.9% |
|  |  | Costs, in millions | −€ 2.9 | −€ 1.9 | −€ 3.5 | −€ 4.2 | −€ 4.2 |
|  |  | N/100,000 population | −€ 0.8 | −€ 0.4 | −€ 0.7 | −€ 0.8 | −€ 0.8 |
|  |  |  | −6.2% | −3.3% | −5.3% | −5.6% | −5.4% |
|  | **Intervention 1_100_ (100% improvement)** | Fractures | −2409 | −1880 | −2741 | −3522 | −3659 |
|  |  | N/100,000 population | −664 | −434 | −553 | −656 | −655 |
|  |  |  | −14.3% | −9.6% | −11.8% | −13.4% | −13.1% |
|  |  | Costs, in millions | −€ 0.3 | € 1.0 | € 0.1 | −€0.8 | −€ 0.8 |
|  |  | N/100,000 population | −€ 0.1 | € 0.2 | € 0.03 | −€ 0.2 | −€ 0.1 |
|  |  |  | −0.7% | 1.8% | 0.2% | −1.1% | −1.1% |
|  | **Intervention 2_100_ (100% improvement)** | Fractures | −2613 | −3234 | −3560 | −4877 | −5232 |
|  |  | N/100,000 population | −720 | −747 | −719 | −908 | −937 |
|  |  |  | −15.5% | −16.5% | −15.4% | −18.6% | −18.7% |
|  |  | Costs, in millions | −€ 3.2 | −€ 3.8 | −€ 3.8 | −€ 5.5 | −€ 5.9 |
|  |  | N/100,000 population | −€ 0.9 | −€ 0.9 | −€ 0.8 | −€ 1,0 | −€ 1.1 |
|  |  |  | −6.6% | −6.8% | −5.8% | −7.4% | −7.6% |
|  | **Intervention 3_100_ (100% improvement)** | Fractures | −3835 | −4085 | −4612 | −5737 | −6339 |
|  |  | N/100,000 population | −1057 | −944 | −931 | −1068 | −1135 |
|  |  |  | −22.7% | −20.9% | −19.9% | −21.8% | −22.6% |
|  |  | Costs, in millions | −€ 0.6 | −€ 0.5 | −€ 0.3 | −€ 1.7 | −€ 2.1 |
|  |  | N/100,000 population | −€ 0.2 | −€ 0.1 | −€ 0.06 | −€ 0.3 | −€ 0.4 |
|  |  |  | −1.2% | −0.8% | −0.5% | −2.3% | −2.7% |
| **Switzerland** |  | | | | | | |
|  | **Intervention 1_10_ (10% improvement)** | Fractures | −2631 | −3293 | −3309 | −3897 | −4340 |
|  |  | N/100,000 population | −395 | −455 | −415 | −433 | −436 |
|  |  |  | −5.2% | −5.8% | −5.2% | −5.5% | −5.5% |
|  |  | Costs, in millions | −€ 27.9 | −€ 35.3 | −€ 35.8 | −€ 42.3 | −€ 47.2 |
|  |  | N/100,000 population | −€ 4.2 | −€ 4.9 | −€ 4.5 | −€ 4.7 | −€ 4.7 |
|  |  |  | −3.7% | −4.3% | −3.9% | −4.1% | −4.1% |
|  | **Intervention 2_10_ (10% improvement)** | Fractures | −2127 | −2450 | −3220 | −3643 | −2742 |
|  |  | N/100,000 population | −320 | −338 | −403 | −405 | −275 |
|  |  |  | −4.2% | −4.3% | −5.1% | −5.2% | −3.5% |
|  |  | Costs, in millions | −€ 23.2 | −€ 26.1 | −€ 34.5 | −€ 39.0 | −€ 28.5 |
|  |  | N/100,000 population | −€ 3.5 | −€ 3.6 | −€ 4.3 | −€ 4.3 | −€ 2.9 |
|  |  |  | −3.1% | −3.2% | −3.8% | −3.8% | −2.5% |
|  | **Intervention 3_10_ (10% improvement)** | Fractures | −3886 | −4493 | −4664 | −5304 | −5938 |
|  |  | N/100,000 population | −584 | −620 | −584 | −590 | −596 |
|  |  |  | −7.6% | −7.9% | −7.4% | −7.5% | −7.5% |
|  |  | Costs, in millions | −€ 42.5 | −€ 49.3 | −€ 51.6 | −€ 58.7 | −€ 65.8 |
|  |  | N/100,000 population | −€ 6.4 | −€ 6.8 | −€ 6.5 | −€ 6.5 | −€ 6.6 |
|  |  |  | −5.7% | −6.0% | −5.6% | −5.7% | −5.7% |
|  | **Intervention 1_50_ (50% improvement)** | Fractures | −6222 | −8224 | −5912 | −6970 | −6880 |
|  |  | N/100,000 population | −935 | −1136 | −741 | −775 | −691 |
|  |  |  | −12.2% | −14.5% | −9.4% | −9.9% | −8.7% |
|  |  | Costs, in millions | −€ 60.6 | −€ 82.1 | −€ 56.2 | −€ 66.8 | −€ 65.0 |
|  |  | N/100,000 population | −€ 9.1 | −€ 11.3 | −€ 7.0 | −€ 7.4 | −€ 6.5 |
|  |  |  | −8.1% | −10.0% | −6.1% | −6.5% | −5.7% |
|  | **Intervention 2_50_ (50% improvement)** | Fractures | −7729 | −8314 | −9232 | −10264 | −11526 |
|  |  | N/100,000 population | −1161 | −1148 | −1156 | −1142 | −1157 |
|  |  |  | −15.2% | −14.7% | −14.6% | −14.5% | −14.6% |
|  |  | Costs, in millions | −€ 86.8 | −€ 92.7 | −€ 102.9 | −€ 114.4 | −€ 128.8 |
|  |  | N/100,000 population | −€ 13.0 | −€ 12.8 | −€ 12.9 | −€ 12.7 | −€ 1.9 |
|  |  |  | −11.7% | −11.3% | −11.2% | −11.1% | −11.2% |
|  | **Intervention 3_50_ (50% improvement)** | Fractures | −14,217 | −17,182 | −17,288 | −19,357 | −20,660 |
|  |  | N/100,000 population | −2136 | −2372 | −2166 | −2153 | −2074 |
|  |  |  | −27.9% | −30.4% | −27.4% | −27.4% | −26.2% |
|  |  | Costs, in millions | −€ 150.8 | −€ 183.1 | −€ 184.9 | −€ 206.9 | −€ 221.0 |
|  |  | N/100,000 population | −€ 22.7 | −€ 25.3 | −€ 23.2 | −€ 23.0 | −€ 22.2 |
|  |  |  | −20.3% | −22.2% | −20.1% | −20.1% | −19.2% |
|  | **Intervention 1_100_ (100% improvement)** | Fractures | −9735 | −9945 | −9878 | −11,321 | −12,884 |
|  |  | N/100,000 population | −1463 | −1373 | −1237 | −1259 | −1293 |
|  |  |  | −19.1% | −17.6% | −15.7% | −16.0% | −16.3% |
|  |  | Costs, in millions | −€ 91.1 | −€ 92.9 | −€ 90.2 | −€ 103.8 | −€ 118.8 |
|  |  | N/100,000 population | −€ 13.7 | −€ 12.8 | −€ 11.3 | −€ 11.5 | −€ 11.9 |
|  |  |  | −12.2% | −11.3% | −9.8% | −10.1% | −10.3% |
|  | **Intervention 2_100_ (100% improvement)** | Fractures | −13,655 | −14,638 | −16,925 | −18,916 | −21,241 |
|  |  | N/100,000 population | −2052 | −2021 | −2120 | −2104 | −2132 |
|  |  |  | −26.8% | −25.9% | −26.8% | −26.8% | −26.9% |
|  |  | Costs, in millions | −€ 151.6 | −€ 162.4 | −€ 187.8 | −€ 209.8 | −€ 235.9 |
|  |  | N/100,000 population | −€ 22.8 | −€ 22.4 | −€ 23.5 | −€ 23.3 | −€ 23.7 |
|  |  |  | −20.4% | −19.7% | −20.4% | −20.4% | −20.5% |
|  | **Intervention 3_100_ (100% improvement)** | Fractures | −19,410 | −19,625 | −22,244 | −25,042 | −28,296 |
|  |  | N/100,000 population | −2917 | −2709 | −2786 | −2785 | −2840 |
|  |  |  | −38.1% | −34.7% | −35.3% | −35.5% | −35.9% |
|  |  | Costs, in millions | −€ 183.8 | −€ 187.4 | −€ 213.7 | −€ 240.1 | −€ 271.8 |
|  |  | N/100,000 population | −€ 27.6 | −€ 25.9 | −€ 26.8 | −€ 26.7 | −€ 27.3 |
|  |  |  | −24.7% | −22.7% | −23.3% | −23.4% | −23.7% |
| **UK** |  | | | | | | |
|  | **Intervention 1_10_ (10% improvement)** | Fractures | −11,614 | −12,798 | −20,776 | −27,708 | −22,666 |
|  |  | N/100,000 population | −229 | −234 | −351 | −428 | −328 |
|  |  |  | −3.8% | −3.8% | −5.6% | −6.8% | −5.2% |
|  |  | Costs, in millions | −€ 65.5 | −€ 72.4 | −€ 123.5 | −€ 167.8 | −€ 133.8 |
|  |  | N/100,000 population | −€ 1.3 | −€ 1.3 | −€ 2.1 | −€ 2.6 | −€ 1.9 |
|  |  |  | −3.0% | −3.0% | −4.7% | −5.8% | −4.3% |
|  | **Intervention 2_10_ (10% improvement)** | Fractures | −14,277 | −15,644 | −9,958 | −15,636 | −11,384 |
|  |  | N/100,000 population | −281 | −286 | −168 | −241 | −165 |
|  |  |  | −4.6% | −4.6% | −2.7% | −3.8% | −2.6% |
|  |  | Costs, in millions | −€ 92.1 | −€ 101.0 | −€ 64.2 | −€ 101.6 | −€ 73.6 |
|  |  | N/100,000 population | −€ 1.8 | −€ 1.8 | −€ 1.1 | −€ 1.6 | −€ 1.1 |
|  |  |  | −4.2% | −4.2% | −2.4% | −3.5% | −2.4% |
|  | **Intervention 3_10_ (10% improvement)** | Fractures | −21,643 | −23,699 | −32,592 | −40,831 | −38,252 |
|  |  | N/100,000 population | −426 | −433 | −551 | −631 | −553 |
|  |  |  | −7.0% | −7.0% | −8.8% | −10.0% | −8.8% |
|  |  | Costs, in millions | −€ 131.1 | −€ 143.7 | −€ 200.8 | −€ 253.6 | −€ 235.8 |
|  |  | N/100,000 population | −€ 2.6 | −€ 2.6 | −€ 3.4 | −€ 3.9 | −€ 3.4 |
|  |  |  | −6.0% | −6.0% | −7.6% | −8.8% | −7.7% |
|  | **Intervention 1_50_ (50% improvement)** | Fractures | −31,168 | −21,710 | −28,134 | −36,062 | −31,155 |
|  |  | N/100,000 population | −613 | −396 | −476 | −557 | −451 |
|  |  |  | −10.1% | −6.4% | −7.6% | −8.9% | −7.2% |
|  |  | Costs, in millions | −€ 151.7 | −€ 86.0 | −€ 123.4 | −€ 169.6 | −€ 133.0 |
|  |  | N/100,000 population | −€ 3.0 | −€ 1.6 | −€ 2.1 | −€ 2.6 | −€ 1.9 |
|  |  |  | −6.9% | −3.6% | −4.7% | −5.9% | −4.3% |
|  | **Intervention 2_50_ (50% improvement)** | Fractures | −44,343 | −49,151 | −43,946 | −51,392 | −51,072 |
|  |  | N/100,000 population | −872 | −898 | −743 | −794 | −739 |
|  |  |  | −14.4% | −14.5% | −11.8% | −12.6% | −11.7% |
|  |  | Costs, in millions | −€ 288.5 | −€ 319.7 | −€ 286.3 | −€ 335.2 | −€ 332.9 |
|  |  | N/100,000 population | −€ 5.7 | −€ 5.8 | −€ 4.8 | −€ 5.2 | −€ 4.8 |
|  |  |  | −13.2% | −13.3% | −10.9% | −11.6% | −10.8% |
|  | **Intervention 3_50_ (50% improvement)** | Fractures | −64,677 | −63,092 | −76,628 | −90,273 | −87,919 |
|  |  | N/100,000 population | −1273 | −1152 | −1296 | −1394 | −1272 |
|  |  |  | −21.0% | −18.6% | −20.6% | −22.2% | −20.2% |
|  |  | Costs, in millions | −€ 370.4 | −€ 356.3 | −€ 440.2 | −€ 523.7 | −€ 503.8 |
|  |  | N/100,000 population | −€ 7.3 | −€ 6.5 | −€ 7.4 | −€ 8.1 | −€ 7.3 |
|  |  |  | −16.9% | −14.8% | −16.7% | −18.2% | −16.4% |
|  | **Intervention 1_100_ (100% improvement)** | Fractures | −41,907 | −46,616 | −55,914 | −54,642 | −66,332 |
|  |  | N/100,000 population | −825 | −851 | −945 | −844 | −960 |
|  |  |  | −13.6% | −13.7% | −15.0% | −13.4% | −15.3% |
|  |  | Costs, in millions | −€ 169.7 | −€ 192.3 | −€ 244.0 | −€ 225.0 | −€ 291.6 |
|  |  | N/100,000 population | −€ 3.3 | −€ 3.5 | −€ 4.1 | −€ 3.5 | −€ 4.2 |
|  |  |  | −7.8% | −8.0% | −9.3% | −7.8% | −9.5% |
|  | **Intervention 2_100_ (100% improvement)** | Fractures | −70,584 | −78,428 | −80,011 | −91,447 | −93,900 |
|  |  | N/100,000 population | −1389 | −1432 | −1353 | −1412 | −1359 |
|  |  |  | −22.9% | −23.1% | −21.5% | −22.5% | −21.6% |
|  |  | Costs, in millions | −€ 459.0 | −€ 510.0 | −€ 521.1 | −€ 596.0 | −€ 611.7 |
|  |  | N/100,000 population | −€ 9.0 | −€ 9.3 | −€ 8.8 | −€ 9.2 | −€ 8.8 |
|  |  |  | −21.0% | −21.2% | −19.8% | −20.7% | −19.9% |
|  | **Intervention 3_100_ (100% improvement)** | Fractures | −92,904 | −103,626 | −114,723 | −118,296 | −135,112 |
|  |  | N/100,000 population | −1828 | −1892 | −1940 | −1827 | −1955 |
|  |  |  | −30.1% | −30.5% | −30.8% | −29.1% | −31.1% |
|  |  | Costs, in millions | −€ 496.1 | −€ 557.2 | −€ 619.6 | −€ 634.1 | −€ 730.9 |
|  |  | N/100,000 population | −€ 9.8 | −€ 10.2 | −€ 10.5 | −€ 9.8 | −€ 10.6 |
|  |  |  | −22.7% | −23.2% | −23.5% | −22.0% | −23.7% |

^a^Reduction in costs of care per 100,000 population (or % reduction) from the status quo.

n/100,000 population is based on women aged ≥70 years.

**References**

1. Lewiecki EM, Ortendahl JD, Vanderpuye-Orgle J, Grauer A, Arellano J, Lemay J, Harmon AL, Broder MS, Singer AJ (2019) Healthcare Policy Changes in Osteoporosis Can Improve Outcomes and Reduce Costs in the United States. JBMR Plus 3:e10192

2. McCloskey E, Rathi J, Heijmans S, et al. (2021) The osteoporosis treatment gap in patients at risk of fracture in European primary care: a multi-country cross-sectional observational study. Osteoporos Int 32:251–259

3. Hernlund E, Svedbom A, Ivergård M, Compston J, Cooper C, Stenmark J, McCloskey EV, Jönsson B, Kanis JA (2013) Osteoporosis in the European Union: medical management, epidemiology and economic burden. A report prepared in collaboration with the International Osteoporosis Foundation (IOF) and the European Federation of Pharmaceutical Industry Associations (EFPIA). Arch Osteoporos 8:136

4. Barrionuevo P, Kapoor E, Asi N, et al. (2019) Efficacy of Pharmacological Therapies for the Prevention of Fractures in Postmenopausal Women: A Network Meta-Analysis. J Clin Endocrinol Metab 104:1623–1630

5. Fardellone P, Barnieh L, Quignot N, Gusto G, Kahangire DA, Worth G, O'Kelly J, Khachatryan A, Desamericq G (2021) The clinical and economic burden after an osteoporosis fracture in France: a nationwide population-based study. Arch Osteoporos 16:124

6. Kanis JA, Borgstrom F, Compston J, Dreinhofer K, Nolte E, Jonsson L, Lems WF, McCloskey EV, Rizzoli R, Stenmark J (2013) SCOPE: a scorecard for osteoporosis in Europe. Arch Osteoporos 8:144

7. Lippuner K, Golder M, Greiner R (2005) Epidemiology and direct medical costs of osteoporotic fractures in men and women in Switzerland. Osteoporos Int 16 Suppl 2:S8-s17

8. Borgström F, Karlsson L, Ortsäter G, et al. (2020) Fragility fractures in Europe: burden, management and opportunities. Arch Osteoporos 15:59

9. Eurostat Population Projections. Available at <https://ec.europa.eu/eurostat/web/population-demography-migration-projections/population-projections-/database> Accessed 04 May 2022.

10. Office of National Statistics. Population Projections. <https://www.ons.gov.uk/peoplepopulationandcommunity/populationandmigration/populationprojections>. Accessed 04 May 2022.
